# Editorial: Next-generation prebiotics and probiotics: Current status and future development

**DOI:** 10.3389/fnut.2022.1023011

**Published:** 2022-09-14

**Authors:** Qingsen Shang

**Affiliations:** ^1^Key Laboratory of Marine Drugs of Ministry of Education, Shandong Provincial Key Laboratory of Glycoscience and Glycotechnology, School of Medicine and Pharmacy, Ocean University of China, Qingdao, China; ^2^Qingdao Marine Biomedical Research Institute, Qingdao, China

**Keywords:** next-generation prebiotics, next-generation probiotics, *Bacillus licheniformis* DSM5749, polydextrose, zymosan, *Euglena gracilis*

Prebiotics and probiotics are widely consumed to maintain health and treat disease all over the world. Although traditional prebiotics and probiotics are easily available in the market, the search for new next-generation prebiotics and probiotics is still urgently needed to enhance their efficacy and health promoting effects. Recently, next-generation prebiotics (for example, mannanoligosaccharide and xylooligosaccharide) and probiotics (for example, *Akkermansia muciniphila* and butyrate-producing bacteria) have emerged in both the academic literature and industry market. In particular, research into next-generation prebiotics and probiotics has tremendously developed. However, still we are at the very beginning to understand how next-generation prebiotics and probiotics work to promote health. The beneficial mechanisms of next-generation prebiotics and probiotics have not been fully elucidated and extensive studies are crucially warranted. In this light, we introduce this Research Topic to advance our understanding of next-generation prebiotics and probiotics.

This Research Topic aims to contribute to filling the gap in the knowledge about the beneficial mechanisms of next-generation prebiotics and probiotics. Specifically, we would like to advance our understanding in the following areas: (1) how next-generation prebiotics work to promote the growth of beneficial microbes and a healthy microbiota in the gut; (2) what effects next-generation prebiotics and probiotics have on the gut microbiota during treatment of diseases; (3) how next-generation prebiotics and probiotics work to promote intestinal health; (4) new advancements in the development of next-generation prebiotics from natural resources; (5) isolation and characterization of next-generation probiotics from human gut or other resources. We would like to bring the latest findings and discoveries in this research field, and we hope that more detailed investigations would be conducted to develop next-generation prebiotics and probiotics as a new class of functional foods or therapeutic drugs.

This editorial summarizes the contributions to the Frontiers Research Topic “*Next-generation prebiotics and probiotics: Current status and future development*.” This Research Topic contains two review articles. Feng et al. analyzed and summarized the effects of probiotics on chemotherapy-induced diarrhea and oral mucositis. They found that orally administrated probiotics could potentially decrease the incidences of chemotherapy-induced diarrhea and oral mucositis. However, more clinical studies are needed to verify this effect and the species and number of probiotics must be optimized and standardized. Huang et al. focused on the effects of probiotics on respiratory tract allergic disease including allergic rhinitis and allergic asthma. They summarized the role of probiotics as an alternative treatment for common allergic diseases in respiratory tract. By reshaping the gut microbiota, probiotics could reduce the allergen-induced hyperreactivity and cytokine release in the respiratory tract. They concluded that it is promising to treat allergic rhinitis and allergic asthma with probiotics. However, they also noted that more detailed future studies must be conducted to solidify the therapeutic effects of probiotics on respiratory tract allergic diseases.

Four original research articles were included in this Research Topic. Among the four research articles, one of them were about next-generation probiotics while the other three were about next-generation prebiotics. Pan et al. investigated the probiotic effects of *Bacillus licheniformis* DSM5749 on the growth performance and gut microbiota of laying hens. They found that *B. licheniformis* DSM5749 could improve the laying performance and promote the intestinal health of hens. Additionally, *B. licheniformis* DSM5749 could increase the abundance of beneficial bacteria (e.g., *Prevotella*) while decrease the abundance of potential pathogenic bacteria (e.g., *Desulfovibrio*) in the gut. This study makes *B. licheniformis* DSM5749 a good candidate for the development of next-generation probiotics. Dai et al. explored the prebiotic effects of different *Euglena gracilis* powders. Using an *in vitro* model, they illustrated that *Euglena gracilis* powders could significantly promote the growth of *Lactobacillus hordei* MRS 102, *Lactobacillus kefiri* MRS 103, *Lactobacillus brevis* MRS 104, *Lactobacillus parabuchneri* MRS 107, *Lactobacillus buchneri* MRS 108 and *Lactobacillus fructivorans* MRS 109. Zhu et al. investigated the potential beneficial effect of polydextrose on the human gut microbiota. They found that adding polydextrose to inulin could reduce the gas production while maintaining the bifidogenic effect. Further study suggested that polydextrose could promote the growth of beneficial bacteria including *Faecalibacterium* spp. and *Roseburia* spp. in the human gut. Similarly, Pi et al. investigated the effect of zymosan on the human gut microbiota and found that it could significantly increase the population of beneficial bacteria such as *Bifidobacterium, Faecalibacterium* and *Prevotella* in the gut. Moreover, zymosan also remarkably increased the gut microbiota production of H_2_ and short chain fatty acids including acetic acid and propionic acid. Collectively, these studies make *Euglena gracilis* powders, polydextrose and zymosan promising candidates for the development of next-generation prebiotics.

This Research Topic provides new insights into the study of next-generation prebiotics and probiotics. As have mentioned above, we are still at the very beginning to understand how next-generation prebiotics and probiotics work to promote health. This Research Topic provides a good beginning in this field and we anticipate that more studies could be conducted to further explore the next-generation prebiotics and probiotics.

## Author contributions

The author confirms being the sole contributor of this work and has approved it for publication.

## Funding

This work was supported by the National Natural Science Foundation of China (32101032), Natural Science Foundation of Shandong Province (ZR2021QC110), and Qingdao Marine Biomedical Research Institute Project (HYJK2021006).

## Conflict of interest

The author declares that the research was conducted in the absence of any commercial or financial relationships that could be construed as a potential conflict of interest.

## Publisher's note

All claims expressed in this article are solely those of the authors and do not necessarily represent those of their affiliated organizations, or those of the publisher, the editors and the reviewers. Any product that may be evaluated in this article, or claim that may be made by its manufacturer, is not guaranteed or endorsed by the publisher.

